# Total hip arthroplasty performed by direct anterior approach – Does experience influence the learning curve?

**DOI:** 10.1051/sicotj/2020015

**Published:** 2020-06-03

**Authors:** Constant Foissey, Mathieu Fauvernier, Cam Fary, Elvire Servien, Sébastien Lustig, Cécile Batailler

**Affiliations:** 1 Department of Orthopaedic Surgery and Sport Medicine, Croix-Rousse Hospital, FIFA Medical Center of Excellence 69004 Lyon France; 2 Department of Biostatistic, Lyon Sud Hospital, University of Lyon 1 69622 Lyon France; 3 Department of Orthopaedic Surgery, University of Melbourne 3010 Victoria Australia; 4 EA 7424, Interuniversity Laboratory of Human Movement Science, Université Lyon 1 69622 Lyon France; 5 Université de Lyon, Université Claude Bernard Lyon 1, IFSTTAR, LBMC UMR_T9406 69622 Lyon France

**Keywords:** Total hip arthroplasty, Direct anterior approach, Learning curve, Complications, Senior surgeon

## Abstract

*Introduction*: Proficiency in the direct anterior approach (DAA) as with many surgical techniques is considered to be challenging. Added to this is the controversy of the benefits of DAA compared to other total hip arthroplasty (THA) approaches. Our study aims to assess the influence of experience on learning curve and clinical results when transitioning from THA via posterior approach in a lateral position to DAA in a supine position. *Methods*: A consecutive retrospective series of 525 total hip arthroplasty of one senior and six junior surgeons was retrospectively analysed from May 2013 to December 2017. Clinical results were analysed and compared between the two groups and represented as a learning curve. Mean follow up was 36.2 months ± 11.8. *Results*: This study found a significant difference in complications between the senior and junior surgeons for operating time, infection rate, and lateral femoral cutaneous nerve (LFCN) neuropraxia. A trainee’s learning curve was an average of 10 DAA procedures before matching the senior surgeon. Of note, the early complications correlated with intraoperative fractures increased with experience in both groups. Operating time for the senior equalised after 70 cases. Dislocation rate and limb length discrepancy were excellent and did not show a learning curve between the two groups. *Conclusion*: DAA is a safe approach to implant a THA. There is a learning curve and initial supervision is recommended for both seniors and trainees. *Level of evidence*: Retrospective, consecutive case series; level IV.

## Introduction

Soft tissue protecting approaches are becoming the gold standard not just in THA and orthopaedics but in the general practise of surgery of all specialties. Hueter followed by Judet [1] first described the direct anterior approach (DAA) in the 50s. Several surgeons modified their approach with or without a traction table [2]. It is described as an anatomical approach [3] leading to a rapid recovery [4, 5], less dislocation [6] allowing a good control of the leg length discrepancy (LLD) [7]. Publications report a lack of evidence for these benefits and significant learning difficulties associated with DAA [8, 9]. Aggarwal et al. [10] compared five surgical approaches and found that DAA had a significantly higher rate of complications (46.8%), as well as re-operations (4.74%). The higher number of re-operations group was due to infection and is in agreement with other authors [11]. Other papers also demonstrated a higher risk of intra-operative fracture in osteoporotic patients [12] and neurological injury [13].

As Zawadsky et al. [14] reported, we do believe that these complications were captured during the learning curve (LC) of the DAA or as Woolson et al. [15] described among inexperienced surgeons without support. Studies have reported the LC of the DAA as 16–88 for the number of DAA to have an acceptable risk of revision [16–19].

Our study aimed to report the clinical results when transitioning from posterior to anterior approach, and to compare the learning curve of a senior surgeon to supervised trainees and outline the more difficult skills to acquire and the duration to acquire equivalent outcomes.

## Material and methods

Five hundred and twenty five THA performed on 474 patients were included retrospectively from May 2013 to December 2017 in a university training hospital. Three hundred and sixty one THA were performed by a senior surgeon and 164 by six junior surgeons (mean = 27 patients [19–35] ± 8) with the same experience (five years of residency learning PA) supervised by the senior. The senior surgeon had performed the DAA technique for one year before supervising the trainees. The accompaniment consisted in teaching tips and tricks, planning together the interventions, validating per-operative control X-ray, and being available in case of difficulties. The LCs of the two groups were analysed and compared. The inclusion criteria were: all primary THA via DAA patients for all surgeons. In our university DAA is standard, except for BMI ≥ 40, complex THA requiring corrective osteotomy (e.g., congenital hip dysplasia, corrective LLD), over 85 yo with osteoporosis, or previous femoral and/or pelvic osteotomy.

### Surgical technique

The standardised approach of Hueter Gaine was used for all patients. The DAA was performed in supine position without an extension table as described by Lustig [20]. Fluoroscopic control was systematically realised during the surgery. A variety of acetabular shells cementless cups were implanted (Dynacup (Tornier^®^), Quattro (Lepine^®^), Pinnacle (Depuy^®^), Tornier DM (Tornier^®^)). Dual mobility cups were used for patients older than 65 yo, and in those with high risk of dislocation (e.g., epilepsy, Parkinson disease, substance abuse). All femoral stems were cementless with a hydroxyapatite coating and of similar design (Corail (Depuy^®^), Meije (Tornier^®^), Targos (Lepine^®^)).

### Patients

Both groups were comparable and the demographic data are presented in Table 1. At follow-up, 2.3% patients died (*n* = 12) (unrelated to THA), 4.8% were lost to follow-up (*n* = 25) Systematic reviews were completed at two months, one year, two years, then every four years with a clinical examination and a radiography. Every patient with a follow-up lower than two years was called back. Mean follow up was 36.2 months ± 11.8 [24–73.4].

**Table 1:** Patient and demographic data.

Parameters	Total	Group senior	Group trainee
THA	488	341	147
Gender (%M)	203 (42%)	139 (41%)	64 (44%)
Mean age (years)			
Mean ± SD [min; max]	65 ± 12 [18; 88]	66 ± 12 [18; 88]	65 ± 13 [30; 87]
Mean BMI (kg/m^2^)			
Mean ± SD [min; max]	26 ± 4 [17; 44]	26 ± 4 [17; 44]	26 ± 4 [17; 36]
Etiology (%)			
Primitive	380 (78%)	262 (77%)	118 (80%)
ONFH	60 (12%)	42 (12%)	18 (12%)
Dysplasia	10 (2%)	9 (3%)	1 (1%)
DDH	18 (3%)	15 (4%)	3 (2%)
Other	20 (5%)	13 (6%)	7 (5%)
Mean pre-op HHS			
Mean ± SD [min; max]	50.5 ± 10 [6; 83]	52 ± 10 [7; 83]	47 ± 11 [6; 72]

THA: total hip arthroplasty, M: male, BMI: body mass index, ONFH: osteonecrosis of the femoral head, DDH: developmental dysplasia of the hip, HHS: Harris hip score, SD: standard deviation.

### Clinical examination

During the pre-operative consultation, we obtained the demographics data, pre-operative Harris hip score (HHS) and the neck shaft angle (NSA).

Operative time, per and post-operative complications were documented as an inpatient.

At follow-up: post-operative HHS, patient satisfaction, clinical leg length discrepancy (LLD), early and late complications were recorded and an anteroposterior and profile pelvic X-ray was taken. The radiographic parameters were assessed at each consultation, but are not reported in this study. Indeed, the aim of this study concerned only the clinical results, clinical complications and revisions.

### Learning curve

LCs were calculated for the senior surgeon and the trainee group of surgeons.

The primary outcome was time to first major or minor complication (MMC) including intraoperative (e.g., femoral fracture) and post-operative complications (e.g., dislocations, infections, ilio-psoas impingement (IPI), lateral femoral cutaneous nerve (LFCN) neuropraxia). The hazard rate associated with MMC was modelled using a flexible hazard regression model [21] and a penalised tensor product spline of MMC and number of operations performed. The data were analysed with survPen [22] from R software [23]. Intra and post-operative complications were analysed individually.

### Statistics

XLSTAT was used for the calculations (version 2015.1, Addinsoft, France). The continuous variables were averaged; Student *t*-test was used to compare quantitative data. Variables were compared using a Fisher exact test or a Chi square test. The learning curves were built with biostatisticians of our department.

Operating time was represented using logarithmic and linear curves for the senior surgeon. This analysis was only performed for the senior group as juniors did not operate enough patients. Level of significance was 5% for every test.

### Ethics approval

All procedures performed in studies involving human participants were in accordance with the ethical standards of the institutional and/or national research committee and with the 1964 Helsinki declaration and its later amendments or comparable ethical standards. The Advisory Committee on Research Information Processing in the Field of Health (CCTIRS) approved this study on June 4, 2015 under number 15–430. For this type of study formal consent is not required.

## Results

### Perioperative complications

**Figure 1:**
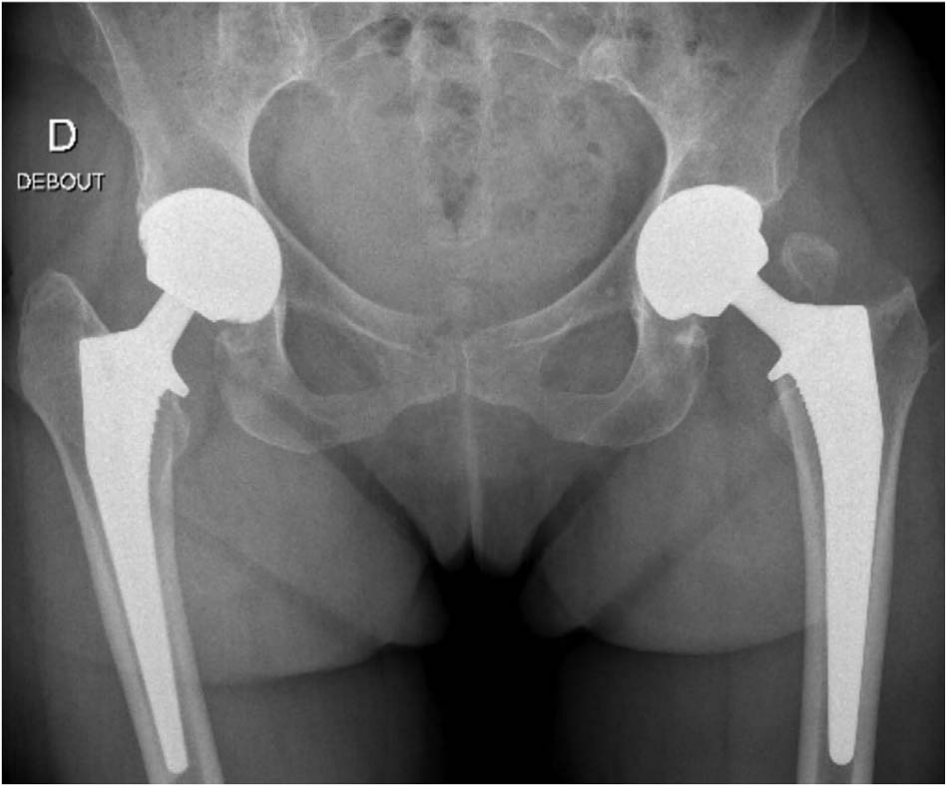
Bilateral two stages THA (Cargos^®^ acetabular cup, Targos^®^ femoral stem, Groupe Lepine^®^, France) with per-operative GT fracture one the left side.

There were 12 greater trochanteric (GT) fractures (2.5%). Two involved the entire GT (Figure 1) and the others were minor superior GT fractures (chip fracture). No GT fractures required fixation, two developed radiological non-union. No GT fracture had any later clinical consequence.

There were five peri-prosthetic fractures (PFF) (1%) at the calcar level. One was fixed intraoperatively with a cable wire, one was non weight bearing for one-month, full weight bearing was allowed for the three others.

The proximal femoral cortex was perforated by the broach twice. Once realised, the perforation was bypassed by the stem and the patient was fully weight bearing post-operatively without any issue.

There were no diaphyseal femoral fractures and there was no significant difference in fractures between the two groups.

### Post-operative complications

Complications are summarised in Table 2.

**Table 2:** Per and postoperative complications.

	Total	Group senior	Group trainee	*p*
No. of hips	488	341	147	
Operation time (min)				
Mean ± SD [min; max]	80 ± 18 [34; 172]	74 ± 14 [34; 133]	93 ± 20 [38; 172]	<0.01*
Peroperative complications (%)	18 (3.7%)	14 (4.1%)	4 (2.7%)	
Greater trochanteric fractures	11 (2.3%)	8 (2.3%)	3 (2%)	1.00
Femoral perforation	1 (0.2%)	1 (0.3%)	0	1.00
PFF	5 (1%)	4 (1.2%)	1 (0.7%)	1.00
Postoperative complications (%)	44(8.6%)	26 (7.8%)	18 (12.7%)	
Dislocation	1 (0.2%)	1 (0.3%)	0	0.30
Infection	8 (1.6%)	1 (0.3%)	7 (4.8%)	0.01*
Aseptic loosening	3 (0.6%)	3 (0.8%)	0	0.56
IPI/psoas pain	22 (4.5%)	18 (5.3%)	4 (2.7%)	0.21
Medius gluteus tendinitis	10 (2%)	8 (2.3%)	2 (1.4%)	0.73
LFCN neuropraxia	12 (2.5%)	2 (0.6%)	10 (6.8%)	<0.01*
LLD unacceptable				
Mean LLD (mm)	7 (1.4%)	4 (1.2%)	3 (2%)	0.43
Mean ± SD [min; max]	0.4 ± 1.8 [−1; 19]	0.3 ± 1.5 [−1; 16]	0.6 ± 2.3 [−1; 19]	0.41
Revision (%)	19 (3.9%)	10 (2.9%)	9 (6.1%)	0.095
Revision with implant removal (%)	8 (1.6%)	4 (1.2%)	4 (2.7%)	0.25

SD: standard deviation, PFF: peri-prosthetic fracture, IPI: iliopsoas impingement, LFCN: lateral femorocutaneous nerve, LLD: leg length discrepancy.

* Significant.

One dislocation occurred in the series when the patient was transferred from the operating table while under anaesthesia onto the bed. This was immediately reduced without any consequence or recurrence in the future.

There were eight deep infections (1.6%), of which one (0.3%) was by the senior surgeon. Three infections were acute and successfully managed with DAIR (Debridement, Antibiotics, and Implant Retention) without recurrence. There were five late deep infections of which all required single stage replacement, three were cured with a further two still being treated.

Twenty-two patients had an IPI or psoas pain. Two patients required acetabular revision (due to lack of cup anteversion) associated with a tenotomy. Three others required a tenotomy under arthroscopy and seven local anaesthetic and steroid injections only.

The LFCN neuropraxias were paraesthesia only, without neurological pain or neuroma development.

Trainee surgeons had significantly more infections and LFCN neuropraxia. There was no difference between the two groups for the IPI.

No implant was more impacted than another by specific complications.

### Clinical outcomes

HHS was significantly improved when compared to pre-operative values: mean postoperative score was 96.2 (±8 [42–100]). 92.4% of the patients were satisfied or very satisfied by the surgery. No significant difference was found between the two groups according to the clinical outcomes.

### Learning curve (Figure 2)

**Figure 2:**
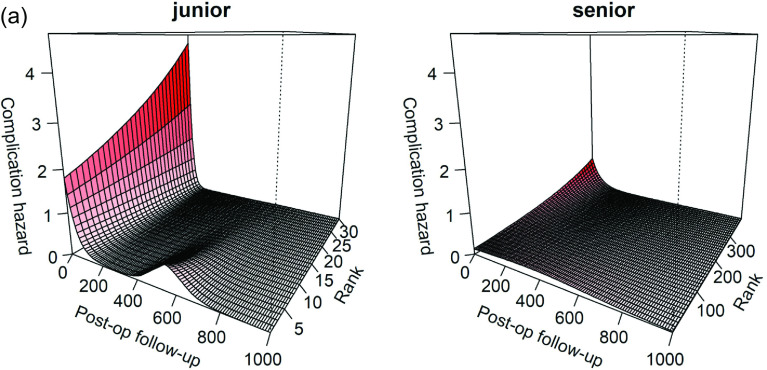
Learning curves. (a) Complication hazard for trainee and senior according to the rank (number of THA) and the time of follow-up (in days). The trainee surgeons had a spike of complications for their 10 first patients at 500 days post-operatively. (b) Comparison of cumulative probability of post-operative complication at 800 days between senior and trainees. The learning curve of the trainee surgeons is faster that of the senior. The trainee surgeons joint the learning curve of the senior after 10 THA. (c) Learning curve of operating time in senior group, with a steady state around 70th case for the senior surgeon. Rank = number of patients operated (experience).

Post-operative complications decreased as experience increased in both groups. The trainee surgeons initially had a spike of complications correlated with the infections described above (Figure 2a). For the 20 first patients, the post-operative complications rates were 20% in both junior and senior groups. For the 20 last patients of this series, the post-operative complications rates were 5% in the junior group and 10% in the senior group.

Paradoxically in both groups, intraoperative complications increased with experience. For the 20 first patients, the per-operative complications rates were 5% in both junior and senior groups. For the 20 last patients of this series, the per-operative complications rates were 20% in the junior group and 10% in the senior group.

The two learning curves are compared on Figure 2b: trainees reached senior skills after 10 DAA THA. The learning curve of junior surgeons improved faster than the senior surgeon.

The LC of operating time (Figure 2c) normalised after 70 cases for the senior surgeon.

## Discussion

This study reported significant differences in the learning curves of both senior and trainee surgeons using DAA to perform THA. The learning curve involved significant changes with operating time, infection rate, and LFCN neuropraxia. This is the first study to compare the LC of experienced and trainee surgeons. In the literature, some studies assessed the learning curve of senior or junior surgeons, but without comparison [16, 19].

The increased rate of infection and neuropraxia in the junior series are associated with a longer operating time and an increased difficulty of exposing the femur with increased soft tissue tension [13]. In literature, the rate of septic implant failures during the DAA learning curve is also significant, and can reach a rate of 2% [16]. LFCN neuropraxia is difficult to assess in literature because not all studies report this and the rate varies between 0.5% and 14.8% [24, 25].

Despite the initial higher rates of complications at the beginning of learning curve for trainees they reached the senior surgeons outcomes after 10 cases. The learning curve of the trainee surgeons improved more quickly than the senior’s. This may be attributed to the senior surgeon supervising and teaching tips and tricks to the trainees. Regardless of surgical approach used, adequate exposure is central to appropriately inserting the prosthesis. Adequate supervision and teaching of trainees is crucial to minimise the learning curve.

It is surprising that in both groups intraoperative complications, predominantly GT fractures, increased with experience. During the initial LC, surgeons avoided operating on the more complex patients (coxa vara, obese, and elderly population) until they felt proficient with the DAA technique. Later, as more challenging patients were operated, adequate exposure became progressively more difficult and bone became more osteoporotic, explaining the paradoxical increase of intraoperative GT fractures. There were no specific complications during the follow-up linked to those fractures. In literature, our increase in intraoperative fractures with increasing experience has not been reported. Possibly other authors had stable indications that did not change rather than increasing complexity as proficiency increased.

The complete and smaller GT fractures (2.5%) that occurred are one of the disadvantages of the DAA. They occur when there is insufficient soft-tissue release requiring increased retractor force to externalise the femur. Several studies reported this complication with a rate varying between 1% and 5.7% during the learning curve of DAA. Two studies tried to identify the risk factors of GT fractures: Homma et al. [26] only found an increased risk of deep trochanters when Hartford et al. [27] found more complications for patients with worse preoperative ambulatory status, diagnosis of SCFE or rheumatoid arthritis, lower femoral neck cut ration, and greater DORR ratio.

No shaft fracture has been reported in our series as part of the LC. Woolson et al. [15] reported that among inexperienced surgeons it was the main complication with 6.5% of femoral shaft or GT fracture. Tay et al. [28] undertook a comparison between anterior, lateral, and posterior approach: anterior approach appeared to be the approach with the higher rate of complications (3.1%), mostly due to PFF (1.4%). The low rate of PFF in this study (1%) could be explained by the non-use of an extension table which is known to put more constraint on the femur.

Some studies described acetabular complications (perforation or fracture of the acetabular floor) [15, 19]. In this study, no intraoperative acetabular complication occurred; we believe that this can be attributed to a combination of routine intraoperative radiograph during the surgery and those over 85 yo with osteoporosis being excluded.

Our results of low dislocation rate for DAA are in line with other studies, Tsukada and Wakui [6] and Sariali et al. [29]. IPI was also related with former series, Ala Eddine et al. [30] reported 4.3% using posterior approach. At the beginning of his LC, the senior surgeon noticed a high rate of IPI. He decided then to put more anteversion in his following cups and to modify his operating technique avoiding the use of an anterior retractor on the anterior horn. The trainees then started their own LC with a full knowledge of this fact, this is why we explain such a lower rate of IPI (2.7%, *n* = 4) compared to the senior (5.3%, *n* = 18).

Lee and Marconi [31] undertook in 2015 a systematic review of 11,810 hips, concerning complications in DAA and found similar results with 2.3% of intraoperative fracture, 2.1% of LFCN neuropraxia, 0.6% of deep infections, and 1.2% of revision.

Equalisation of leg length was uncomplicated. Bingham et al. [32] reported no difference in leg length inequality between the use or not of the fluoroscopy in DAA.

The total complication rate of this study (15%) is similar to other studies on THA by DAA, but there is a wide variation from 9% to 44% in the learning curves [33]. Woolson et al. reported a 9% incidence of major complications in a group of community orthopaedic surgeons in their learning curve with the DAA [15]. Kong et al. reported an initial higher rate of complications of 44% in the first 50 cases of the learning curve which decreased to 16% in the second 50 cases [17].

A steady state of operating time was reached at around 70 cases for the senior which is similar to literature [17]. This improvement of the operating time is attributed to a reduction in fluoroscopy time and a more efficient exposure.

No study describes the LC for posterior or lateral approach; thus it is very difficult to compare approaches. LC of the DAA is the only approach to have such an analysis in the literature. In our institution and others LC analysis of other approaches is difficult as exposure to them by trainees occurs early and is mostly on traumatic hip fractures with osteoporotic bone. Meta-analysis by Miller et al. [34] comparing DAA versus posterior approach found a significant lower rate of infection, dislocation, and reoperation in favour of DAA. Conversely a higher rate of nerve injury was found using DAA. However, these comparisons were made after the learning curve.

Our study demonstrates that complications cannot be analysed as a single entity without taking the learning curve and technical complexity of the individual patient into account: as the LC evolves so can the type of complication (intra-operative vs. post-operative). Using our method, no statistically rational rank could be calculated accurately. Several papers studied LCs with different methods, but usually these methods were approximative, with a rank varying between 16 and 88 [16–19]. Usually studies determined the rank of learning curve like the rank where the complications rate begins approximately to decrease.

This study had several limits. Firstly, there were multiple implants which have their own learning curve for implantation which can affect complications and operating time [35]. However, the implants were of equivalent characteristics and designs. This is a retrospective study. However, the aims of this study were to assess early complication and revision rates, which are not influenced by retrospective analysis.

## Conclusion

The results obtained in this study allow us to conclude that DAA is a safe approach to implant a THA. However, as with any surgical technique, a LC has to be taken into account and a surgeon experienced in the technique is highly advisable for training when learning.
